# 2,2′-(Hexane-1,6-di­yl)diisoquinolinium tetra­chloridozincate(II)

**DOI:** 10.1107/S1600536809017693

**Published:** 2009-05-20

**Authors:** Pei-Hua Ma, Zhi-Fang Fan, Yun-Qian Zhang, Xin Xiao, Sai-Feng Xue

**Affiliations:** a Key Laboratory of Macrocyclic and Supramolecular Chemistry of Guizhou Province, Guizhou University, Guiyang 550025, People’s Republic of China; b Institute of Applied Chemistry, Guizhou University, Guiyang 550025, People’s Republic of China

## Abstract

The asymmetric unit of the title compound, (C_24_H_26_N_2_)[ZnCl_4_], consists of two 2,2′-(hexane-1,6-di­yl)diisoquinolinium cations and two [ZnCl_4_]^2−^ complex anions. The [ZnCl_4_]^2−^ anions have a distorted tetra­hedral geometry. The dihedral angles between the isoquinoline rings of the two cations are nearly equal [16.1 (2) and 16.3 (2)°]. In the crystal structure, the ordered linear formation is aggregated by weak inter­molecular π–π stacking inter­actions between neighboring isoquinoline pyridine rings with a centroid–centroid distance of 3.779 (4) Å.

## Related literature

For general background to quinoline compounds, see: Day & Arnold (2000 ▶); Day *et al.* (2002 ▶); Freeman *et al.* (1981 ▶); Kim *et al.* (2000 ▶); Wu *et al.* (2008 ▶). For a related structure, see: Pan & Xu (2004 ▶).
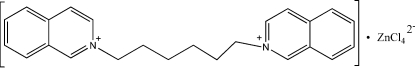


## Experimental

### 

#### Crystal data

(C_24_H_26_N_2_)[ZnCl_4_]
*M* *_r_* = 549.66
Monoclinic, 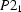

*a* = 10.066 (2) Å
*b* = 24.666 (5) Å
*c* = 10.392 (2) Å
β = 108.826 (2)°
*V* = 2442.2 (8) Å^3^
*Z* = 4
Mo *K*α radiation
μ = 1.46 mm^−1^
*T* = 293 K
0.24 × 0.22 × 0.19 mm

#### Data collection

Bruker SMART CCD area-detector diffractometer
Absorption correction: multi-scan (*SADABS*; Bruker, 2005 ▶) *T*_min_ = 0.708, *T*_max_ = 0.755
11146 measured reflections
8189 independent reflections
6898 reflections with *I* > 2σ(*I*)
*R*_int_ = 0.027

#### Refinement

*R*[*F* ^2^ > 2σ(*F* ^2^)] = 0.041
*wR*(*F* ^2^) = 0.101
*S* = 1.05
8189 reflections
559 parameters
1 restraint
H-atom parameters constrained
Δρ_max_ = 0.53 e Å^−3^
Δρ_min_ = −0.27 e Å^−3^
Absolute structure: Flack (1983 ▶), 3771 Friedel pairs
Flack parameter: 0.263 (12)

### 

Data collection: *SMART* (Bruker, 2002 ▶); cell refinement: *SAINT* (Bruker, 2002 ▶); data reduction: *SAINT*; program(s) used to solve structure: *SHELXS97* (Sheldrick, 2008 ▶); program(s) used to refine structure: *SHELXL97* (Sheldrick, 2008 ▶); molecular graphics: *ORTEP-3 for Windows* (Farrugia, 1997 ▶); software used to prepare material for publication: *WinGX* (Farrugia, 1999 ▶).

## Supplementary Material

Crystal structure: contains datablocks global, I. DOI: 10.1107/S1600536809017693/bq2137sup1.cif

Structure factors: contains datablocks I. DOI: 10.1107/S1600536809017693/bq2137Isup2.hkl

Additional supplementary materials:  crystallographic information; 3D view; checkCIF report

## Figures and Tables

**Table 1:** Selected bond lengths (Å)

Cl1—Zn1	2.2773 (15)
Cl2—Zn1	2.2836 (15)
Cl3—Zn1	2.2674 (16)
Cl4—Zn1	2.3023 (14)
Cl5—Zn2	2.2969 (14)
Cl6—Zn2	2.2826 (16)
Cl7—Zn2	2.2770 (15)
Cl8—Zn2	2.2898 (15)

**Fig. 1.**
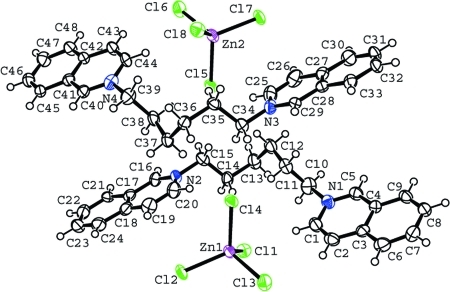
The molecular structure of (I) showing the atom-labeling scheme. Displacement ellipsoids are drawn at the 50% probability level

## supplementary crystallographic information

### Comment

As part of our ongoing investigation on quinoline compounds, we present here the crystal structure of the compound with multiple functional groups, which can develop strong intermolecular interactions with cucurbit[*n*]urils (CB[*n*]) (Freeman *et al.*, 1981; Day & Arnold, 2000; Day *et al.*, 2002; Kim *et al.*, 2000; Wu *et al.*, 2008).

The crystal structure of the title compound (Fig. 1) consists of organic cations and anionic complex [ZnCl_4_]^2-^. The [ZnCl_4_]^2-^ anion is a distorted tetrahedron with Zn–Cl bonds distances ranging from 2.2674 (16)Å to 2.3023 (14)Å (table 1). There are dihedral angles of 16.1 (2)Å between the N1 isoquinolyl ring (N1/C1-C9) and N2 isoquinolyl ring (N2/C16-C24), 16.3 (2)Å between the N3 isoquinolyl ring (N3/C25-C33) and N4 isoquinolyl ring (N4/C40-C48) of organic cations, respectively. The ordered linear formation was aggregated by intermolecular weak π-π stacking interactions between neighboring pyridine rings of isoquinolyl in the crystal structure. (Pan & Xu, 2004).

### Experimental

A solution of 1,6-dibromine-hexane (2.44 g, 0.01 mol) was added to a stirred solution of isoquinoline (2.58 g, 0.02 mol) in 1,4-dioxane (50 ml) at 373 K in a period of 5 h. After cooling to room temperature, the mixture was filtered. The residue was added to an aqueous solution (50 ml) of ZnCl_2_ (0.01 mol, 1.37 g). After stirring for 2 h, the solution was filtered. Colorless single crystals of the title compound were obtained from the filtrate after 3 weeks.

### Refinement

H atoms were placed in calculated positions with C—H = 0.93Å (aromatic) or 0.97Å (methylene), and refined in riding mode with *U*_iso_(H) = 1.2*U*_eq_(C).

### Crystal data

**Table:**

(C_24_H_26_N_2_)[ZnCl_4_]	*F*(000) = 1128
*M**_r_* = 549.66	*D*_x_ = 1.495 Mg m^−^^3^
Monoclinic, *P*2_1_	Mo *K*α radiation, λ = 0.71073 Å
Hall symbol: p 2yb	Cell parameters from 8189 reflections
*a* = 10.066 (2) Å	θ = 1.7–25.0°
*b* = 24.666 (5) Å	µ = 1.46 mm^−^^1^
*c* = 10.392 (2) Å	*T* = 293 K
β = 108.826 (2)°	Prism, colorless
*V* = 2442.2 (8) Å^3^	0.24 × 0.22 × 0.19 mm
*Z* = 4	

### Data collection

**Table:**

Bruker SMART CCD area-detector diffractometer	8189 independent reflections
Radiation source: fine-focus sealed tube	6898 reflections with *I* > 2σ(*I*)
graphite	*R*_int_ = 0.027
φ and ω scans	θ_max_ = 25.0°, θ_min_ = 1.7°
Absorption correction: multi-scan (*SADABS*; Bruker, 2005)	*h* = −11→11
*T*_min_ = 0.708, *T*_max_ = 0.755	*k* = −29→29
11146 measured reflections	*l* = −12→12

### Refinement

**Table:**

Refinement on *F*^2^	Secondary atom site location: difference Fourier map
Least-squares matrix: full	Hydrogen site location: inferred from neighbouring sites
*R*[*F*^2^ > 2σ(*F*^2^)] = 0.041	H-atom parameters constrained
*wR*(*F*^2^) = 0.101	*w* = 1/[σ^2^(*F*_o_^2^) + (0.0514*P*)^2^ + ] where *P* = (*F*_o_^2^ + 2*F*_c_^2^)/3
*S* = 1.05	(Δ/σ)_max_ < 0.001
8189 reflections	Δρ_max_ = 0.53 e Å^−^^3^
559 parameters	Δρ_min_ = −0.27 e Å^−^^3^
1 restraint	Absolute structure: Flack (1983)
Primary atom site location: structure-invariant direct methods	Flack parameter: 0.263 (12)

### Special details

**Table:**

Geometry. All e.s.d.'s (except the e.s.d. in the dihedral angle between two l.s. planes) are estimated using the full covariance matrix. The cell e.s.d.'s are taken into account individually in the estimation of e.s.d.'s in distances, angles and torsion angles; correlations between e.s.d.'s in cell parameters are only used when they are defined by crystal symmetry. An approximate (isotropic) treatment of cell e.s.d.'s is used for estimating e.s.d.'s involving l.s. planes.
Refinement. Refinement of *F*^2^ against ALL reflections. The weighted *R*-factor *wR* and goodness of fit *S* are based on *F*^2^, conventional *R*-factors *R* are based on *F*, with *F* set to zero for negative *F*^2^. The threshold expression of *F*^2^ > σ(*F*^2^) is used only for calculating *R*-factors(gt) *etc*. and is not relevant to the choice of reflections for refinement. *R*-factors based on *F*^2^ are statistically about twice as large as those based on *F*, and *R*- factors based on ALL data will be even larger.

### Fractional atomic coordinates and isotropic or equivalent isotropic displacement parameters (Å^2^)

**Table:**

	*x*	*y*	*z*	*U*_iso_*/*U*_eq_	
C1	0.6424 (7)	0.8906 (2)	0.5696 (6)	0.0438 (16)	
H1	0.7012	0.8939	0.6590	0.053*	
C2	0.5037 (7)	0.8871 (2)	0.5450 (6)	0.0402 (14)	
H2	0.4674	0.8885	0.6167	0.048*	
C3	0.4126 (6)	0.8813 (2)	0.4109 (6)	0.0327 (14)	
C4	0.4749 (6)	0.8794 (2)	0.3035 (6)	0.0331 (13)	
C5	0.6204 (6)	0.8838 (2)	0.3404 (6)	0.0340 (14)	
H5	0.6622	0.8827	0.2726	0.041*	
C6	0.2646 (7)	0.8791 (2)	0.3751 (7)	0.0445 (15)	
H6	0.2228	0.8793	0.4428	0.053*	
C7	0.1821 (6)	0.8766 (2)	0.2405 (6)	0.0449 (15)	
H7	0.0849	0.8766	0.2178	0.054*	
C8	0.2448 (6)	0.8740 (2)	0.1369 (6)	0.0381 (14)	
H8	0.1881	0.8713	0.0466	0.046*	
C9	0.3871 (6)	0.8754 (2)	0.1673 (6)	0.0363 (14)	
H9	0.4265	0.8737	0.0978	0.044*	
C10	0.8554 (6)	0.8982 (3)	0.5011 (7)	0.0489 (18)	
H10A	0.8835	0.8890	0.4229	0.059*	
H10B	0.9052	0.8744	0.5753	0.059*	
C11	0.8960 (6)	0.9567 (2)	0.5418 (6)	0.0413 (14)	
H11A	0.8836	0.9637	0.6290	0.050*	
H11B	0.8333	0.9806	0.4754	0.050*	
C12	1.0468 (6)	0.9698 (3)	0.5517 (6)	0.0444 (16)	
H12A	1.0569	0.9643	0.4628	0.053*	
H12B	1.0637	1.0079	0.5741	0.053*	
C13	1.1580 (6)	0.9373 (3)	0.6536 (5)	0.0459 (16)	
H13A	1.2476	0.9449	0.6413	0.055*	
H13B	1.1381	0.8991	0.6347	0.055*	
C14	1.1717 (5)	0.9480 (3)	0.8037 (5)	0.0375 (13)	
H14A	1.0799	0.9450	0.8147	0.045*	
H14B	1.2318	0.9205	0.8603	0.045*	
C15	1.2317 (7)	1.0033 (2)	0.8507 (6)	0.0423 (15)	
H15A	1.3226	1.0068	0.8379	0.051*	
H15B	1.1702	1.0309	0.7963	0.051*	
C16	1.3714 (6)	1.0060 (2)	1.0920 (6)	0.0343 (14)	
H16	1.4484	0.9960	1.0665	0.041*	
C17	1.3897 (6)	1.0148 (2)	1.2312 (6)	0.0313 (13)	
C18	1.2714 (6)	1.0299 (2)	1.2658 (6)	0.0399 (15)	
C19	1.1421 (6)	1.0346 (2)	1.1615 (7)	0.0464 (16)	
H19	1.0623	1.0443	1.1827	0.056*	
C20	1.1325 (6)	1.0252 (2)	1.0313 (6)	0.0444 (15)	
H20	1.0457	1.0279	0.9638	0.053*	
C21	1.5238 (6)	1.0102 (2)	1.3312 (6)	0.0321 (14)	
H21	1.6016	1.0009	1.3063	0.039*	
C22	1.5376 (7)	1.0196 (2)	1.4624 (6)	0.0445 (15)	
H22	1.6252	1.0156	1.5281	0.053*	
C23	1.4217 (8)	1.0354 (2)	1.5025 (7)	0.0524 (19)	
H23	1.4339	1.0422	1.5936	0.063*	
C24	1.2900 (7)	1.0407 (2)	1.4061 (7)	0.0473 (16)	
H24	1.2139	1.0513	1.4323	0.057*	
C25	0.7866 (6)	0.1287 (3)	0.4333 (6)	0.0461 (15)	
H25	0.8725	0.1260	0.5022	0.055*	
C26	0.7785 (7)	0.1196 (3)	0.3046 (7)	0.0488 (17)	
H26	0.8596	0.1109	0.2845	0.059*	
C27	0.6484 (6)	0.1228 (2)	0.1972 (6)	0.0355 (13)	
C28	0.5293 (6)	0.1381 (2)	0.2328 (6)	0.0297 (13)	
C29	0.5447 (6)	0.1472 (2)	0.3690 (5)	0.0289 (12)	
H29	0.4668	0.1571	0.3932	0.035*	
C30	0.6318 (7)	0.1124 (3)	0.0592 (6)	0.0437 (16)	
H30	0.7086	0.1019	0.0339	0.052*	
C31	0.5034 (7)	0.1177 (2)	−0.0365 (6)	0.0439 (15)	
H31	0.4943	0.1110	−0.1270	0.053*	
C32	0.3841 (6)	0.1330 (2)	−0.0034 (5)	0.0382 (14)	
H32	0.2975	0.1366	−0.0708	0.046*	
C33	0.3975 (6)	0.1422 (2)	0.1286 (6)	0.0346 (13)	
H33	0.3185	0.1516	0.1515	0.041*	
C34	0.6825 (6)	0.1510 (2)	0.6086 (6)	0.0400 (14)	
H34A	0.7411	0.1226	0.6628	0.048*	
H34B	0.5909	0.1485	0.6203	0.048*	
C35	0.7466 (5)	0.2055 (2)	0.6595 (5)	0.0310 (11)	
H35A	0.6895	0.2338	0.6034	0.037*	
H35B	0.8393	0.2075	0.6503	0.037*	
C36	0.7584 (5)	0.2156 (2)	0.8064 (5)	0.0322 (13)	
H36A	0.6688	0.2072	0.8181	0.039*	
H36B	0.7763	0.2539	0.8257	0.039*	
C37	0.8733 (5)	0.1829 (2)	0.9109 (5)	0.0372 (14)	
H37A	0.8563	0.1447	0.8903	0.045*	
H37B	0.8649	0.1891	1.0002	0.045*	
C38	1.0249 (5)	0.1961 (2)	0.9173 (5)	0.0355 (13)	
H38A	1.0890	0.1725	0.9836	0.043*	
H38B	1.0354	0.1887	0.8294	0.043*	
C39	1.0640 (6)	0.2547 (2)	0.9556 (6)	0.0406 (15)	
H39A	1.0155	0.2781	0.8798	0.049*	
H39B	1.0342	0.2646	1.0324	0.049*	
C40	1.2976 (6)	0.2689 (2)	1.1181 (6)	0.0328 (13)	
H40	1.2561	0.2702	1.1861	0.039*	
C41	1.4448 (6)	0.2733 (2)	1.1543 (6)	0.0280 (13)	
C42	1.5065 (6)	0.2720 (2)	1.0519 (6)	0.0313 (12)	
C43	1.4151 (6)	0.2673 (2)	0.9159 (6)	0.0360 (14)	
H43	1.4528	0.2670	0.8451	0.043*	
C44	1.2763 (6)	0.2631 (2)	0.8872 (6)	0.0376 (14)	
H44	1.2187	0.2603	0.7973	0.045*	
C45	1.5328 (6)	0.2760 (2)	1.2922 (6)	0.0327 (13)	
H45	1.4931	0.2763	1.3616	0.039*	
C46	1.6741 (6)	0.2781 (2)	1.3229 (6)	0.0358 (14)	
H46	1.7313	0.2805	1.4131	0.043*	
C47	1.7329 (6)	0.2767 (2)	1.2202 (6)	0.0422 (14)	
H47	1.8301	0.2778	1.2427	0.051*	
C48	1.6543 (6)	0.2737 (2)	1.0885 (6)	0.0379 (15)	
H48	1.6972	0.2728	1.0216	0.045*	
Cl1	0.88950 (14)	0.88576 (5)	0.91266 (15)	0.0409 (3)	
Cl2	0.81540 (16)	0.97308 (6)	1.18102 (13)	0.0441 (4)	
Cl3	0.52795 (15)	0.91812 (7)	0.89273 (15)	0.0475 (4)	
Cl4	0.76150 (15)	1.02597 (5)	0.82706 (13)	0.0355 (3)	
Cl5	0.16104 (15)	0.12917 (5)	0.63448 (13)	0.0358 (3)	
Cl6	0.38644 (15)	0.24126 (7)	0.56637 (15)	0.0461 (4)	
Cl7	0.10464 (16)	0.18099 (6)	0.28203 (13)	0.0444 (4)	
Cl8	0.02407 (14)	0.26899 (6)	0.54912 (14)	0.0413 (3)	
N1	0.7007 (5)	0.88939 (18)	0.4672 (5)	0.0365 (11)	
N2	1.2473 (5)	1.01179 (18)	0.9969 (5)	0.0343 (11)	
N3	0.6674 (5)	0.14215 (18)	0.4643 (5)	0.0339 (11)	
N4	1.2176 (5)	0.26308 (18)	0.9914 (5)	0.0363 (12)	
Zn1	0.74988 (6)	0.95121 (2)	0.95564 (6)	0.03348 (16)	
Zn2	0.16703 (6)	0.20425 (2)	0.50571 (6)	0.03468 (16)	

### Atomic displacement parameters (Å^2^)

**Table:**

	*U*^11^	*U*^22^	*U*^33^	*U*^12^	*U*^13^	*U*^23^
C1	0.058 (4)	0.040 (3)	0.031 (4)	−0.001 (3)	0.011 (3)	0.001 (3)
C2	0.049 (4)	0.038 (3)	0.037 (4)	0.001 (3)	0.018 (3)	0.003 (3)
C3	0.042 (4)	0.022 (3)	0.033 (4)	−0.002 (3)	0.010 (3)	0.004 (2)
C4	0.040 (3)	0.030 (3)	0.030 (3)	−0.002 (2)	0.012 (3)	−0.001 (2)
C5	0.041 (4)	0.039 (3)	0.026 (4)	0.002 (3)	0.016 (3)	0.001 (3)
C6	0.055 (4)	0.041 (3)	0.046 (4)	−0.006 (3)	0.028 (3)	−0.004 (3)
C7	0.040 (4)	0.044 (4)	0.049 (4)	−0.007 (3)	0.012 (3)	−0.006 (3)
C8	0.050 (4)	0.036 (3)	0.025 (3)	−0.003 (3)	0.008 (3)	0.001 (3)
C9	0.052 (4)	0.035 (3)	0.025 (3)	0.001 (3)	0.016 (3)	−0.001 (3)
C10	0.034 (4)	0.053 (4)	0.057 (5)	0.010 (3)	0.011 (3)	0.002 (3)
C11	0.044 (4)	0.046 (4)	0.032 (3)	0.005 (3)	0.010 (3)	−0.004 (3)
C12	0.054 (4)	0.050 (4)	0.031 (4)	0.001 (3)	0.016 (3)	−0.004 (3)
C13	0.033 (3)	0.072 (5)	0.034 (4)	−0.003 (3)	0.013 (3)	−0.004 (3)
C14	0.024 (3)	0.054 (3)	0.031 (3)	−0.004 (3)	0.004 (2)	−0.003 (3)
C15	0.052 (4)	0.044 (4)	0.026 (3)	−0.001 (3)	0.006 (3)	0.005 (3)
C16	0.031 (3)	0.030 (3)	0.039 (4)	−0.002 (2)	0.007 (3)	−0.007 (3)
C17	0.037 (3)	0.021 (3)	0.040 (4)	−0.002 (2)	0.018 (3)	−0.005 (2)
C18	0.046 (4)	0.030 (3)	0.045 (4)	−0.009 (3)	0.016 (3)	−0.006 (3)
C19	0.038 (4)	0.049 (4)	0.057 (4)	0.003 (3)	0.023 (3)	−0.007 (3)
C20	0.032 (3)	0.053 (4)	0.041 (4)	−0.003 (3)	0.001 (3)	−0.007 (3)
C21	0.043 (4)	0.027 (3)	0.027 (3)	−0.004 (2)	0.013 (3)	0.004 (2)
C22	0.048 (4)	0.032 (3)	0.047 (4)	−0.005 (3)	0.006 (3)	−0.001 (3)
C23	0.086 (6)	0.038 (4)	0.037 (4)	−0.015 (4)	0.026 (4)	−0.004 (3)
C24	0.052 (4)	0.050 (4)	0.047 (4)	−0.010 (3)	0.027 (4)	−0.009 (3)
C25	0.036 (4)	0.050 (4)	0.045 (4)	0.001 (3)	0.003 (3)	−0.003 (3)
C26	0.032 (4)	0.054 (4)	0.062 (5)	−0.007 (3)	0.018 (3)	−0.012 (4)
C27	0.038 (3)	0.034 (3)	0.036 (3)	−0.010 (3)	0.014 (3)	−0.007 (3)
C28	0.036 (3)	0.022 (3)	0.028 (3)	−0.003 (2)	0.006 (3)	0.002 (2)
C29	0.037 (3)	0.026 (3)	0.022 (3)	−0.002 (2)	0.007 (3)	0.003 (2)
C30	0.051 (4)	0.047 (4)	0.041 (4)	−0.006 (3)	0.026 (3)	−0.011 (3)
C31	0.066 (5)	0.042 (4)	0.022 (3)	−0.009 (3)	0.013 (3)	−0.006 (3)
C32	0.046 (4)	0.034 (3)	0.023 (3)	−0.002 (3)	−0.005 (3)	0.004 (2)
C33	0.041 (3)	0.022 (3)	0.037 (4)	0.005 (2)	0.006 (3)	−0.003 (2)
C34	0.048 (4)	0.041 (3)	0.025 (3)	−0.006 (3)	0.005 (3)	0.001 (3)
C35	0.031 (3)	0.036 (3)	0.024 (3)	0.000 (2)	0.006 (2)	0.005 (3)
C36	0.029 (3)	0.035 (3)	0.031 (3)	−0.004 (2)	0.008 (2)	−0.005 (2)
C37	0.033 (3)	0.048 (3)	0.026 (3)	−0.010 (3)	0.004 (3)	−0.003 (3)
C38	0.030 (3)	0.042 (3)	0.026 (3)	0.000 (3)	−0.002 (2)	0.000 (3)
C39	0.023 (3)	0.045 (4)	0.046 (4)	0.001 (2)	0.000 (3)	0.000 (3)
C40	0.037 (3)	0.025 (3)	0.035 (4)	−0.001 (2)	0.009 (3)	−0.004 (3)
C41	0.033 (3)	0.023 (3)	0.027 (3)	0.001 (2)	0.008 (3)	0.001 (2)
C42	0.040 (3)	0.028 (3)	0.027 (3)	−0.002 (2)	0.012 (3)	−0.002 (2)
C43	0.045 (4)	0.040 (3)	0.023 (3)	−0.002 (3)	0.011 (3)	−0.001 (3)
C44	0.042 (4)	0.045 (3)	0.017 (3)	−0.004 (3)	−0.003 (3)	0.003 (3)
C45	0.046 (4)	0.025 (3)	0.025 (3)	−0.001 (2)	0.008 (3)	0.000 (2)
C46	0.035 (3)	0.039 (3)	0.031 (4)	−0.001 (3)	0.007 (3)	−0.003 (3)
C47	0.034 (3)	0.045 (3)	0.041 (4)	0.005 (3)	0.003 (3)	0.004 (3)
C48	0.036 (3)	0.048 (4)	0.030 (4)	0.002 (3)	0.012 (3)	0.001 (3)
Cl1	0.0365 (8)	0.0378 (8)	0.0471 (9)	0.0038 (6)	0.0118 (7)	0.0051 (7)
Cl2	0.0472 (9)	0.0663 (10)	0.0171 (7)	−0.0117 (7)	0.0082 (7)	−0.0044 (6)
Cl3	0.0333 (8)	0.0687 (10)	0.0425 (9)	−0.0064 (7)	0.0152 (7)	−0.0095 (8)
Cl4	0.0477 (9)	0.0298 (7)	0.0268 (7)	0.0028 (6)	0.0089 (7)	0.0055 (6)
Cl5	0.0482 (9)	0.0368 (7)	0.0206 (7)	0.0020 (6)	0.0087 (6)	0.0039 (6)
Cl6	0.0305 (8)	0.0726 (10)	0.0371 (9)	−0.0096 (7)	0.0133 (7)	−0.0084 (8)
Cl7	0.0476 (9)	0.0655 (10)	0.0196 (7)	−0.0127 (7)	0.0101 (7)	−0.0041 (7)
Cl8	0.0319 (8)	0.0489 (8)	0.0412 (9)	0.0032 (6)	0.0092 (7)	0.0054 (7)
N1	0.039 (3)	0.038 (3)	0.032 (3)	0.006 (2)	0.010 (2)	0.002 (2)
N2	0.038 (3)	0.036 (3)	0.024 (3)	−0.001 (2)	0.003 (2)	−0.001 (2)
N3	0.034 (3)	0.032 (3)	0.030 (3)	−0.002 (2)	0.003 (2)	−0.002 (2)
N4	0.026 (3)	0.030 (3)	0.048 (3)	0.003 (2)	0.004 (2)	−0.002 (2)
Zn1	0.0339 (4)	0.0408 (4)	0.0251 (3)	−0.0015 (3)	0.0086 (3)	0.0010 (3)
Zn2	0.0316 (4)	0.0468 (4)	0.0246 (4)	−0.0016 (3)	0.0077 (3)	0.0014 (3)

### Geometric parameters (Å, °)

**Table:**

C1—C2	1.339 (8)	C26—H26	0.9300
C1—N1	1.371 (7)	C27—C30	1.413 (8)
C1—H1	0.9300	C27—C28	1.414 (7)
C2—C3	1.407 (8)	C28—C29	1.392 (7)
C2—H2	0.9300	C28—C33	1.420 (7)
C3—C6	1.415 (8)	C29—N3	1.316 (6)
C3—C4	1.446 (8)	C29—H29	0.9300
C4—C5	1.393 (7)	C30—C31	1.359 (8)
C4—C9	1.410 (8)	C30—H30	0.9300
C5—N1	1.312 (7)	C31—C32	1.403 (8)
C5—H5	0.9300	C31—H31	0.9300
C6—C7	1.378 (8)	C32—C33	1.354 (8)
C6—H6	0.9300	C32—H32	0.9300
C7—C8	1.415 (8)	C33—H33	0.9300
C7—H7	0.9300	C34—N3	1.474 (7)
C8—C9	1.364 (8)	C34—C35	1.510 (7)
C8—H8	0.9300	C34—H34A	0.9700
C9—H9	0.9300	C34—H34B	0.9700
C10—N1	1.497 (7)	C35—C36	1.514 (7)
C10—C11	1.521 (9)	C35—H35A	0.9700
C10—H10A	0.9700	C35—H35B	0.9700
C10—H10B	0.9700	C36—C37	1.535 (7)
C11—C12	1.522 (7)	C36—H36A	0.9700
C11—H11A	0.9700	C36—H36B	0.9700
C11—H11B	0.9700	C37—C38	1.541 (7)
C12—C13	1.501 (8)	C37—H37A	0.9700
C12—H12A	0.9700	C37—H37B	0.9700
C12—H12B	0.9700	C38—C39	1.519 (8)
C13—C14	1.543 (7)	C38—H38A	0.9700
C13—H13A	0.9700	C38—H38B	0.9700
C13—H13B	0.9700	C39—N4	1.483 (7)
C14—C15	1.508 (8)	C39—H39A	0.9700
C14—H14A	0.9700	C39—H39B	0.9700
C14—H14B	0.9700	C40—N4	1.311 (7)
C15—N2	1.491 (7)	C40—C41	1.411 (7)
C15—H15A	0.9700	C40—H40	0.9300
C15—H15B	0.9700	C41—C42	1.395 (7)
C16—N2	1.327 (7)	C41—C45	1.423 (7)
C16—C17	1.414 (8)	C42—C48	1.412 (7)
C16—H16	0.9300	C42—C43	1.421 (7)
C17—C18	1.401 (8)	C43—C44	1.334 (7)
C17—C21	1.418 (7)	C43—H43	0.9300
C18—C19	1.404 (8)	C44—N4	1.391 (7)
C18—C24	1.434 (8)	C44—H44	0.9300
C19—C20	1.345 (8)	C45—C46	1.355 (7)
C19—H19	0.9300	C45—H45	0.9300
C20—N2	1.356 (7)	C46—C47	1.378 (7)
C20—H20	0.9300	C46—H46	0.9300
C21—C22	1.345 (8)	C47—C48	1.344 (8)
C21—H21	0.9300	C47—H47	0.9300
C22—C23	1.414 (8)	C48—H48	0.9300
C22—H22	0.9300	Cl1—Zn1	2.2773 (15)
C23—C24	1.386 (9)	Cl2—Zn1	2.2836 (15)
C23—H23	0.9300	Cl3—Zn1	2.2674 (16)
C24—H24	0.9300	Cl4—Zn1	2.3023 (14)
C25—C26	1.333 (8)	Cl5—Zn2	2.2969 (14)
C25—N3	1.379 (7)	Cl6—Zn2	2.2826 (16)
C25—H25	0.9300	Cl7—Zn2	2.2770 (15)
C26—C27	1.422 (8)	Cl8—Zn2	2.2898 (15)
			
C2—C1—N1	122.1 (6)	N3—C29—C28	121.3 (5)
C2—C1—H1	118.9	N3—C29—H29	119.4
N1—C1—H1	118.9	C28—C29—H29	119.4
C1—C2—C3	120.1 (6)	C31—C30—C27	119.9 (6)
C1—C2—H2	120.0	C31—C30—H30	120.0
C3—C2—H2	120.0	C27—C30—H30	120.0
C2—C3—C6	123.9 (5)	C30—C31—C32	122.2 (5)
C2—C3—C4	117.5 (6)	C30—C31—H31	118.9
C6—C3—C4	118.5 (5)	C32—C31—H31	118.9
C5—C4—C9	123.0 (5)	C33—C32—C31	118.7 (5)
C5—C4—C3	117.7 (5)	C33—C32—H32	120.6
C9—C4—C3	119.3 (5)	C31—C32—H32	120.6
N1—C5—C4	122.5 (5)	C32—C33—C28	121.6 (5)
N1—C5—H5	118.8	C32—C33—H33	119.2
C4—C5—H5	118.8	C28—C33—H33	119.2
C7—C6—C3	120.5 (5)	N3—C34—C35	112.4 (4)
C7—C6—H6	119.8	N3—C34—H34A	109.1
C3—C6—H6	119.8	C35—C34—H34A	109.1
C6—C7—C8	120.2 (6)	N3—C34—H34B	109.1
C6—C7—H7	119.9	C35—C34—H34B	109.1
C8—C7—H7	119.9	H34A—C34—H34B	107.9
C9—C8—C7	121.0 (5)	C34—C35—C36	112.7 (4)
C9—C8—H8	119.5	C34—C35—H35A	109.1
C7—C8—H8	119.5	C36—C35—H35A	109.1
C8—C9—C4	120.4 (5)	C34—C35—H35B	109.1
C8—C9—H9	119.8	C36—C35—H35B	109.1
C4—C9—H9	119.8	H35A—C35—H35B	107.8
N1—C10—C11	111.7 (5)	C35—C36—C37	115.0 (4)
N1—C10—H10A	109.3	C35—C36—H36A	108.5
C11—C10—H10A	109.3	C37—C36—H36A	108.5
N1—C10—H10B	109.3	C35—C36—H36B	108.5
C11—C10—H10B	109.3	C37—C36—H36B	108.5
H10A—C10—H10B	107.9	H36A—C36—H36B	107.5
C10—C11—C12	113.1 (5)	C36—C37—C38	115.4 (5)
C10—C11—H11A	109.0	C36—C37—H37A	108.4
C12—C11—H11A	109.0	C38—C37—H37A	108.4
C10—C11—H11B	109.0	C36—C37—H37B	108.4
C12—C11—H11B	109.0	C38—C37—H37B	108.4
H11A—C11—H11B	107.8	H37A—C37—H37B	107.5
C13—C12—C11	115.8 (5)	C39—C38—C37	112.5 (5)
C13—C12—H12A	108.3	C39—C38—H38A	109.1
C11—C12—H12A	108.3	C37—C38—H38A	109.1
C13—C12—H12B	108.3	C39—C38—H38B	109.1
C11—C12—H12B	108.3	C37—C38—H38B	109.1
H12A—C12—H12B	107.4	H38A—C38—H38B	107.8
C12—C13—C14	115.2 (5)	N4—C39—C38	111.1 (4)
C12—C13—H13A	108.5	N4—C39—H39A	109.4
C14—C13—H13A	108.5	C38—C39—H39A	109.4
C12—C13—H13B	108.5	N4—C39—H39B	109.4
C14—C13—H13B	108.5	C38—C39—H39B	109.4
H13A—C13—H13B	107.5	H39A—C39—H39B	108.0
C15—C14—C13	112.2 (5)	N4—C40—C41	121.9 (5)
C15—C14—H14A	109.2	N4—C40—H40	119.1
C13—C14—H14A	109.2	C41—C40—H40	119.1
C15—C14—H14B	109.2	C42—C41—C40	118.9 (5)
C13—C14—H14B	109.2	C42—C41—C45	119.0 (5)
H14A—C14—H14B	107.9	C40—C41—C45	122.1 (5)
N2—C15—C14	110.9 (4)	C41—C42—C48	118.9 (5)
N2—C15—H15A	109.5	C41—C42—C43	117.1 (5)
C14—C15—H15A	109.5	C48—C42—C43	123.9 (5)
N2—C15—H15B	109.5	C44—C43—C42	121.6 (5)
C14—C15—H15B	109.5	C44—C43—H43	119.2
H15A—C15—H15B	108.0	C42—C43—H43	119.2
N2—C16—C17	121.7 (5)	C43—C44—N4	120.2 (5)
N2—C16—H16	119.1	C43—C44—H44	119.9
C17—C16—H16	119.1	N4—C44—H44	119.9
C18—C17—C16	117.5 (5)	C46—C45—C41	120.3 (5)
C18—C17—C21	121.3 (5)	C46—C45—H45	119.9
C16—C17—C21	121.1 (5)	C41—C45—H45	119.9
C17—C18—C19	118.5 (6)	C45—C46—C47	119.8 (6)
C17—C18—C24	117.9 (6)	C45—C46—H46	120.1
C19—C18—C24	123.6 (6)	C47—C46—H46	120.1
C20—C19—C18	120.6 (6)	C48—C47—C46	122.1 (6)
C20—C19—H19	119.7	C48—C47—H47	119.0
C18—C19—H19	119.7	C46—C47—H47	119.0
C19—C20—N2	121.1 (6)	C47—C48—C42	120.0 (6)
C19—C20—H20	119.4	C47—C48—H48	120.0
N2—C20—H20	119.4	C42—C48—H48	120.0
C22—C21—C17	119.5 (6)	C5—N1—C1	120.2 (5)
C22—C21—H21	120.3	C5—N1—C10	120.6 (5)
C17—C21—H21	120.3	C1—N1—C10	119.1 (5)
C21—C22—C23	121.4 (6)	C16—N2—C20	120.5 (5)
C21—C22—H22	119.3	C16—N2—C15	120.6 (5)
C23—C22—H22	119.3	C20—N2—C15	119.0 (5)
C24—C23—C22	120.0 (6)	C29—N3—C25	121.6 (5)
C24—C23—H23	120.0	C29—N3—C34	121.0 (5)
C22—C23—H23	120.0	C25—N3—C34	117.5 (5)
C23—C24—C18	119.9 (6)	C40—N4—C44	120.3 (5)
C23—C24—H24	120.0	C40—N4—C39	121.3 (5)
C18—C24—H24	120.0	C44—N4—C39	118.4 (5)
C26—C25—N3	119.9 (6)	Cl3—Zn1—Cl1	107.53 (6)
C26—C25—H25	120.0	Cl3—Zn1—Cl2	108.20 (5)
N3—C25—H25	120.0	Cl1—Zn1—Cl2	112.11 (6)
C25—C26—C27	121.4 (6)	Cl3—Zn1—Cl4	110.54 (6)
C25—C26—H26	119.3	Cl1—Zn1—Cl4	107.69 (5)
C27—C26—H26	119.3	Cl2—Zn1—Cl4	110.74 (6)
C30—C27—C28	118.8 (5)	Cl7—Zn2—Cl6	107.83 (6)
C30—C27—C26	124.3 (6)	Cl7—Zn2—Cl8	113.14 (6)
C28—C27—C26	117.0 (5)	Cl6—Zn2—Cl8	106.28 (6)
C29—C28—C27	118.9 (5)	Cl7—Zn2—Cl5	109.94 (6)
C29—C28—C33	122.3 (5)	Cl6—Zn2—Cl5	111.50 (6)
C27—C28—C33	118.8 (5)	Cl8—Zn2—Cl5	108.13 (5)
			
N1—C1—C2—C3	0.8 (9)	C30—C31—C32—C33	−0.5 (9)
C1—C2—C3—C6	−177.9 (5)	C31—C32—C33—C28	1.4 (8)
C1—C2—C3—C4	0.0 (8)	C29—C28—C33—C32	−179.7 (5)
C2—C3—C4—C5	−0.5 (8)	C27—C28—C33—C32	−1.2 (8)
C6—C3—C4—C5	177.6 (5)	N3—C34—C35—C36	178.3 (4)
C2—C3—C4—C9	−178.0 (5)	C34—C35—C36—C37	73.1 (6)
C6—C3—C4—C9	0.1 (8)	C35—C36—C37—C38	64.7 (6)
C9—C4—C5—N1	177.5 (5)	C36—C37—C38—C39	60.8 (6)
C3—C4—C5—N1	0.1 (8)	C37—C38—C39—N4	167.1 (5)
C2—C3—C6—C7	176.2 (6)	N4—C40—C41—C42	0.8 (7)
C4—C3—C6—C7	−1.7 (8)	N4—C40—C41—C45	−175.7 (5)
C3—C6—C7—C8	2.6 (8)	C40—C41—C42—C48	−176.9 (5)
C6—C7—C8—C9	−1.8 (8)	C45—C41—C42—C48	−0.3 (8)
C7—C8—C9—C4	0.1 (8)	C40—C41—C42—C43	1.0 (7)
C5—C4—C9—C8	−176.6 (5)	C45—C41—C42—C43	177.7 (5)
C3—C4—C9—C8	0.7 (8)	C41—C42—C43—C44	−1.2 (8)
N1—C10—C11—C12	−169.4 (5)	C48—C42—C43—C44	176.6 (6)
C10—C11—C12—C13	−60.5 (7)	C42—C43—C44—N4	−0.4 (8)
C11—C12—C13—C14	−67.0 (7)	C42—C41—C45—C46	0.9 (8)
C12—C13—C14—C15	−70.1 (7)	C40—C41—C45—C46	177.5 (5)
C13—C14—C15—N2	−178.4 (5)	C41—C45—C46—C47	−1.1 (8)
N2—C16—C17—C18	0.0 (8)	C45—C46—C47—C48	0.6 (9)
N2—C16—C17—C21	−177.9 (5)	C46—C47—C48—C42	0.1 (9)
C16—C17—C18—C19	1.0 (7)	C41—C42—C48—C47	−0.2 (8)
C21—C17—C18—C19	178.8 (5)	C43—C42—C48—C47	−178.1 (6)
C16—C17—C18—C24	−177.8 (5)	C4—C5—N1—C1	0.7 (8)
C21—C17—C18—C24	0.1 (8)	C4—C5—N1—C10	−175.3 (5)
C17—C18—C19—C20	−0.5 (9)	C2—C1—N1—C5	−1.2 (9)
C24—C18—C19—C20	178.2 (6)	C2—C1—N1—C10	174.8 (5)
C18—C19—C20—N2	−1.1 (9)	C11—C10—N1—C5	105.5 (6)
C18—C17—C21—C22	1.3 (8)	C11—C10—N1—C1	−70.5 (7)
C16—C17—C21—C22	179.0 (5)	C17—C16—N2—C20	−1.5 (8)
C17—C21—C22—C23	−1.8 (8)	C17—C16—N2—C15	178.9 (5)
C21—C22—C23—C24	1.0 (9)	C19—C20—N2—C16	2.1 (9)
C22—C23—C24—C18	0.4 (9)	C19—C20—N2—C15	−178.3 (5)
C17—C18—C24—C23	−0.9 (8)	C14—C15—N2—C16	100.4 (6)
C19—C18—C24—C23	−179.6 (6)	C14—C15—N2—C20	−79.2 (7)
N3—C25—C26—C27	−0.7 (10)	C28—C29—N3—C25	1.4 (8)
C25—C26—C27—C30	−178.5 (6)	C28—C29—N3—C34	−179.0 (5)
C25—C26—C27—C28	2.3 (9)	C26—C25—N3—C29	−1.2 (9)
C30—C27—C28—C29	178.6 (5)	C26—C25—N3—C34	179.2 (5)
C26—C27—C28—C29	−2.1 (7)	C35—C34—N3—C29	−102.7 (6)
C30—C27—C28—C33	0.1 (8)	C35—C34—N3—C25	77.0 (7)
C26—C27—C28—C33	179.4 (5)	C41—C40—N4—C44	−2.5 (8)
C27—C28—C29—N3	0.3 (7)	C41—C40—N4—C39	175.7 (5)
C33—C28—C29—N3	178.8 (5)	C43—C44—N4—C40	2.3 (8)
C28—C27—C30—C31	0.7 (8)	C43—C44—N4—C39	−176.0 (5)
C26—C27—C30—C31	−178.5 (6)	C38—C39—N4—C40	−104.8 (6)
C27—C30—C31—C32	−0.5 (9)	C38—C39—N4—C44	73.4 (6)
